# Effects of transcranial direct current stimulation (tDCS) at different cortical targets on cognition in obsessive-compulsive disorder (OCD): an exploratory analysis

**DOI:** 10.1097/YIC.0000000000000589

**Published:** 2025-04-04

**Authors:** Luca Pellegrini, Eduardo Cinosi, David Wellsted, Megan Smith, Amanda Busby, Natalie Hall, Umberto Albert, Ibrahim Aslan, Matt Garner, Samuel R. Chamberlain, Trevor W. Robbins, David S. Baldwin, Naomi A. Fineberg

**Affiliations:** a School of Life and Medical Sciences, University of Hertfordshire, Hatfield; b Centre for Neuropsychopharmacology and Psychedelic Research, Hammersmith Hospital Campus, Imperial College, London, UK; c Department of Medicine, Surgery and Health Sciences, University of Trieste; d UCO Clinica Psichiatrica, Azienda Sanitaria Universitaria Giuliano-Isontina – ASUGI, Trieste, Italy; e Hertfordshire Partnership University NHS Foundation Trust, Welwyn Garden City; f Clinical and Experimental Sciences, Faculty of Medicine, University of Southampton; g Hampshire and Isle of Wight Healthcare NHS Foundation Trust, Tatchbury Mount, Southampton; h Department of Psychology and Behavioural and Clinical Neuroscience Institute; i School of Clinical Medicine, University of Cambridge, Cambridge, UK

**Keywords:** cognitive inflexibility, motor impulsivity, obsessive-compulsive disorder, transcranial direct current stimulation

## Abstract

Transcranial direct current stimulation (tDCS) holds promise as a treatment for obsessive-compulsive disorder (OCD). Patients with OCD show impairment in specific domains of cognitive flexibility and response inhibition. We previously reported that tDCS produced a positive clinical effect on OCD symptoms. Here, we report a secondary analysis of neurocognitive data. In this randomized, double-blind, sham-controlled, crossover, multicenter feasibility study, adults with a diagnosis of OCD according to the diagnostic and statistical manual of mental disorders, fifth edition (DSM-5) received three courses of clinic-based tDCS, targeting the left orbitofrontal cortex (L-OFC), bilateral supplementary motor area (SMA), and sham, randomly allocated and delivered in counterbalanced order. Cognitive assessments were conducted before and 2-h after the first stimulation in each arm. Nineteen adults were recruited. tDCS of both the L-OFC and SMA significantly improved cognitive inflexibility, while sham treatment did not (paired-sample *t* test, baseline vs. 2-h after stimulation). No significant effect of tDCS was found for motor impulsivity (stop-signal reaction time) in any of the three arms. In a small sample of patients with OCD, a single administration of tDCS to the L-OFC and SMA produced a rapid improvement in cognitive inflexibility but not in motor impulsivity. A definitive randomized, controlled trial of tDCS targeting both the OFC and SMA, including cognitive markers, is indicated.

## Introduction

Obsessive-compulsive disorder (OCD) is a common mental disorder and a significant contributor to mental health morbidity (Albert *et al*., 2019; Fineberg *et al*., 2020; Benatti *et al*., 2021; Kochar *et al*., 2023). As a sizable minority of OCD patients (roughly 40%) do not experience an adequate response to recommended first-line treatments (Fineberg *et al*., 2020; Varinelli *et al*., 2022), alternatives are under investigation. OCD has a relatively well-defined neuroanatomical basis (Tyagi *et al*., 2019; Soriano-Mas, 2021; Veltman, 2021). Research has therefore been focused on noninvasive forms of brain stimulation, including repetitive transcranial magnetic stimulation (rTMS) (Pellegrini *et al*., 2022) and transcranial direct current stimulation (tDCS) (Fineberg *et al*., 2023) targeting putative OCD-related dysfunctions in fronto-striato-thalamic neuro-circuitry, including the dorsolateral prefrontal cortex (dlPFC), anterior cingulate cortex (ACC), supplementary motor area (SMA), orbitofrontal cortex (OFC), and medial prefrontal cortex (Milad and Rauch, 2011; Gomes *et al.*, 2013; Fineberg *et al*., 2020, 2023).

Based on a small number of preliminary studies, tDCS has shown promise as a safe and effective treatment for OCD (Fineberg *et al*., 2023) with potential for development as a self-administered intervention. tDCS delivers a weak electrical current (typically 1–2 mA) to the scalp, which passes through the brain tissue, causing depolarization or hyperpolarization of neurons in the targeted brain region. This modulation of neuronal activity can lead to changes in brain functioning and behavior. The effects of tDCS can last for several minutes to hours after stimulation.

Several studies have investigated the efficacy of tDCS in OCD. Seven sham-controlled randomized controlled trials (RCTs) have been conducted, with considerable variation in the protocols used. All seven RCTSs (Bation *et al*., 2019; Gowda *et al*., 2019; Yoosefee *et al*., 2020; Silva *et al*., 2021; Adams *et al*., 2022; Balzus *et al*., 2022; Fineberg *et al*., 2023) included patients taking medication for OCD. The results showed an acute positive effect for tDCS on Yale-Brown Obsessive-Compulsive Scale (Y-BOCS) scores, but the effects were short-lived. A meta-analysis of eight studies of tDCS in OCD (Pinto *et al*., 2022), of which four studies were RCTs (Bation *et al*., 2019; Gowda *et al*., 2019; Yoosefee *et al*., 2020; Silva *et al*., 2021) involving a total of 241 individuals (165 active treatment, 76 sham), found that tDCS produced a significant improvement compared with the control condition. The risk of bias in many studies, however, was high and in a secondary analysis that only considered RCTs, active tDCS was no longer superior to sham (Pinto *et al*., 2022).

It is recognized that impulsive or rigid responding are specific aspects of executive dysfunction of relevance to well-being (Clarke *et al*., 2024) and may have adverse consequences on the effectiveness of therapeutic interventions. Individuals with OCD who display inflexible behaviors (Fineberg *et al*., 2015) tend to exhibit greater levels of resistance to therapy (Wetterneck *et al*., 2011), hence increasing their susceptibility to relapse (Eisen *et al*., 2013; Cirnigliaro *et al*., 2021). Moreover, it has been shown that the lack of cognitive flexibility has a detrimental effect on the efficacy of cognitive-behavioral therapy (CBT) (D’Alcante *et al*., 2012; Simpson *et al*., 2021).

The effect of tDCS on OCD-relevant aspects of executive cognitive function has been studied, but only in disorders other than OCD. Soyata *et al*. (2019) investigated 20 patients with gambling disorder, finding that tDCS over the dlPFC resulted in significantly increased advantageous decision-making and cognitive flexibility (measured through the Wisconsin Card Sorting Test). In a study of 25 children with attention deficit hyperactivity disorder (ADHD) (Nejati *et al*., 2020), inhibitory control (measured via a go/no-go task) was improved by cathodal tDCS applied to the dlPFC, while cognitive flexibility and task switching (measured through the Wisconsin Cards Sorting Test) were improved by combined stimulation of the dlPFC and the OFC, but not by dlPFC stimulation alone. Other recent studies have attempted to modulate cognitive inflexibility and executive functioning with tDCS in healthy subjects (Borwick *et al*., 2020), individuals with major depressive disorder (Koshikawa *et al*., 2022), and autism spectrum disorder (Parmar *et al*., 2021), with encouraging but only preliminary results.

We performed a randomized, double-blind, sham-controlled, crossover, multicenter feasibility study (Fineberg et al, 2023), in which 20 adults with DSM-5 OCD received three courses of clinic-based tDCS [bilateral SMA, lateral OFC (L-OFC), sham], randomly allocated and delivered in counterbalanced order. Each course comprised four 20-min 2 mA stimulations, delivered over two consecutive days, separated by a ‘washout’ period of at least 4 weeks. tDCS was found to be safe, acceptable, and well tolerated. The study was not powered to produce a statistically significant difference between treatment arms on symptom severity following intervention (due to it being a relatively small study focusing on feasibility rather than a larger definitive clinical trial to assess efficacy); however, Y-BOCS scores were numerically improved from baseline to 24 h after the final stimulation and the greatest effect size vs. sham was seen in the L-OFC arm, [Cohen’s *d* = −0.5 (95% confidence interval [CI]: −1.2 to 0.2)]. The study additionally tested the feasibility of collecting cognitive outcomes via the Cambridge Neuropsychological Test Automated Battery (CANTAB) (Robbins *et al*., 1998), in particular measures of motor impulsivity [stop-signal reaction time (SSRT)] and cognitive inflexibility [intradimensional, extradimensional set shifting (ID-ED)], which are known as latent cognitive phenotypes of OCD of relevance to well-being (Chamberlain *et al*., 2007; Clarke *et al*., 2024), to determine the effect of tDCS on these OCD-related cognitive mechanisms.

The aim of this element of the study was to investigate the effects of tDCS on cognitive markers of motor inhibition, representing the ability to withhold and cancel inappropriate responses, and cognitive flexibility, representing the ability to shift attention from one perceptual feature or dimension of a stimulus to another potentially more relevant dimension, in patients with OCD. Based on the emerging literature, these specific neurocognitive markers, implicated in the mechanism of OCD (Tyagi *et al*., 2019; Reid *et al*., 2024), could represent theoretically plausible mechanisms to explain the positive clinical effects of tDCS in OCD.

## Methods

### Ethics committee approval

Approval from the Cambridgeshire and Hertfordshire Research Ethics Committee was obtained on 27 March 2019 (reference number 19/EE/0046). Subsequently, the research gained clearance from the Health Research Authority to commence on 29 March 2019.

### Design

This is a secondary analysis of a previously published study (Fineberg *et al*., 2023). In the following section, we summarize key aspects of the methodology to aid interpretation of our findings. Full methodological details are available in the original publication (Fineberg *et al*., 2023). Participants were assigned to receive two courses of active tDCS, targeting both the L-OFC and the bilateral SMA and one course of sham stimulation, following a crossover design. tDCS was allocated in one of six randomized sequences determined using a simple, counterbalanced randomization method. Each course was administered over a span of two consecutive days. Each course was separated by a 28-day interval and comprised four consecutive 20-min, 2 mA stimulations. The stimulation was conducted inside a quiet clinical environment, where the patient remained conscious and seated in a comfortable chair and adhered to safety criteria as outlined by experts (Fregni *et al*., 2015). The participants were kept blinded as to whether they were receiving sham or active stimulation. The outcome evaluations were conducted by researchers who had received training and were fully blinded to the specific tDCS target and the kind of stimulation being administered (active or sham).

### Cognitive assessment

Participants underwent in-person cognitive assessments using selected tasks from the CANTAB Battery (Robbins *et al*., 1998) immediately before (referred to as baseline) and 2 h after (referred to as postbaseline) the first stimulation of each course of tDCS (specific tasks described below).

#### Stop-signal reaction time

Motor impulsivity refers to the inability to control cued actions. We evaluated this using the stop-signal task (SST), providing a sensitive estimate of the amount of time (measured in milliseconds) required to inhibit prepotent motor responses. This paradigm is sensitive to motor impulsivity associated with ADHD, OCD, trichotillomania, and damage to the right inferior frontal gyrus (Aron *et al*., 2004; Chamberlain *et al*., 2006). Participants are asked to respond quickly to left- or right-facing arrows delivered onscreen with corresponding motor responses (left or right buttons) and to endeavor to inhibit responding when an auditory ‘stop signal’ sounds. Using a tracking algorithm, this task measures the time required to suppress prepotent motor responses internally (referred to as the SSRT). The SSRT is the primary metric used to evaluate inhibitory performance on this task. It refers to the amount of time necessary to inhibit the response elicited by the ‘go’ signal in the presence of the ‘withhold’ signal. This is calculated using a tracking algorithm that computes the average response time to ‘go’ trials and the amount of time for which participants are able to successfully withhold the response. Deficiencies in response inhibition correlate with impulsivity. The distinguishing feature of the SST, setting it apart from other assessments of motor impulsivity, is that inhibition occurs after, rather than before, the response starts. The SST paradigm has been successfully translated into nonhuman primates, rats, and mice, revealing notable parallels in the normal values of important variables, such as the SSRT, which support the conclusion that analogous mechanisms are involved in both animal and human research (Robbins *et al*., 2019).

#### Intradimensional/extradimensional set shift task

Cognitive flexibility is the mental capacity to switch between thinking about two distinct concepts or to simultaneously consider multiple concepts. The intradimensional/extradimensional set shift task is a computerized task, adapted from the Wisconsin Card Sorting Task (Grant & Berg, 1948; Milner B., 1963). It tests rule acquisition, rule reversal, attentional shifting, and attentional flexibility via a sequence of staged subtasks. During this test, participants must use trial and error feedback to determine a rule for identifying the correct stimulus among a variety of stimulus pairings. At the beginning of each task stage, the rule for a correct response is modified to dissociate distinct aspects of cognitive flexibility. The intradimensional shift stage evaluates rule generalization when novel stimuli within the same dimension are introduced, while the extradimensional shift (EDS; stage 8) evaluates the capacity to inhibit and shift attention away from a previously relevant stimulus dimension to a different one (similar to a ‘category shift’ on the Wisconsin Card Sorting Test). The EDS is regarded as being the most important for OCD because it probes a vital aspect of cognitive flexibility – the capacity to ‘unlearn’ a previously learnt rule and transfer one’s attention to a new stimulus dimension that was previously irrelevant. We were therefore most interested in performance on the EDS stage, in particular the number of errors made in the process of successfully completing this stage of the task, which in previous studies has demonstrated sensitivity for capturing cognitive inflexibility in OCD (Chamberlain *et al*., 2005; Fineberg *et al*., 2015).

### Statistical analysis

Analysis of the cognitive data was performed using JASP (Version 0.16.3), a freely available statistical program created by the University of Amsterdam (JASP Team, 2024). Analysis of variance (ANOVA) was used to compare differences in mean scores between treatment arms. Assumption checks were performed to assess normality using the Shapiro–Wilk test. Paired samples Student *t* tests were used to examine variations within each treatment arm where normality could be assumed. Specifically, this was accomplished by comparing baseline cognitive test scores with postbaseline scores conducted 2 h after the first stimulation in each modality. In cases where departures from normality were observed, the Wilcoxon signed-rank test was used. Effect sizes of the differences between pre- and posttreatment values of L-OFC, SMA, and sham were determined using Cohen’s *d (d*) or the matched rank biserial correlation (*r*_bs_), as appropriate. Statistical value was set at *P* < 0.05.

## Results

Nineteen participants provided data for this analysis (clinical data was available for 20 subjects, while neurocognitive data was available only for 19 participants that took part in the original study). Participants had a mean age of 45 (SD: 16.6) years; 10 were male and 9 female. Fifteen participants (79%) were taking stable doses of medication: 13 selective serotonin reuptake inhibitor (SSRI) ± adjunctive psychotropic medication, 2 venlafaxine, and 2 other medications. The three treatment groups (L-OFC, SMA, sham) were similarly matched at baseline for sociodemographic and clinical characteristics (for further details of sample characteristics, refer to original article, Fineberg, *et al*., 2023).

### Cognitive performance

**Fig. 1:**
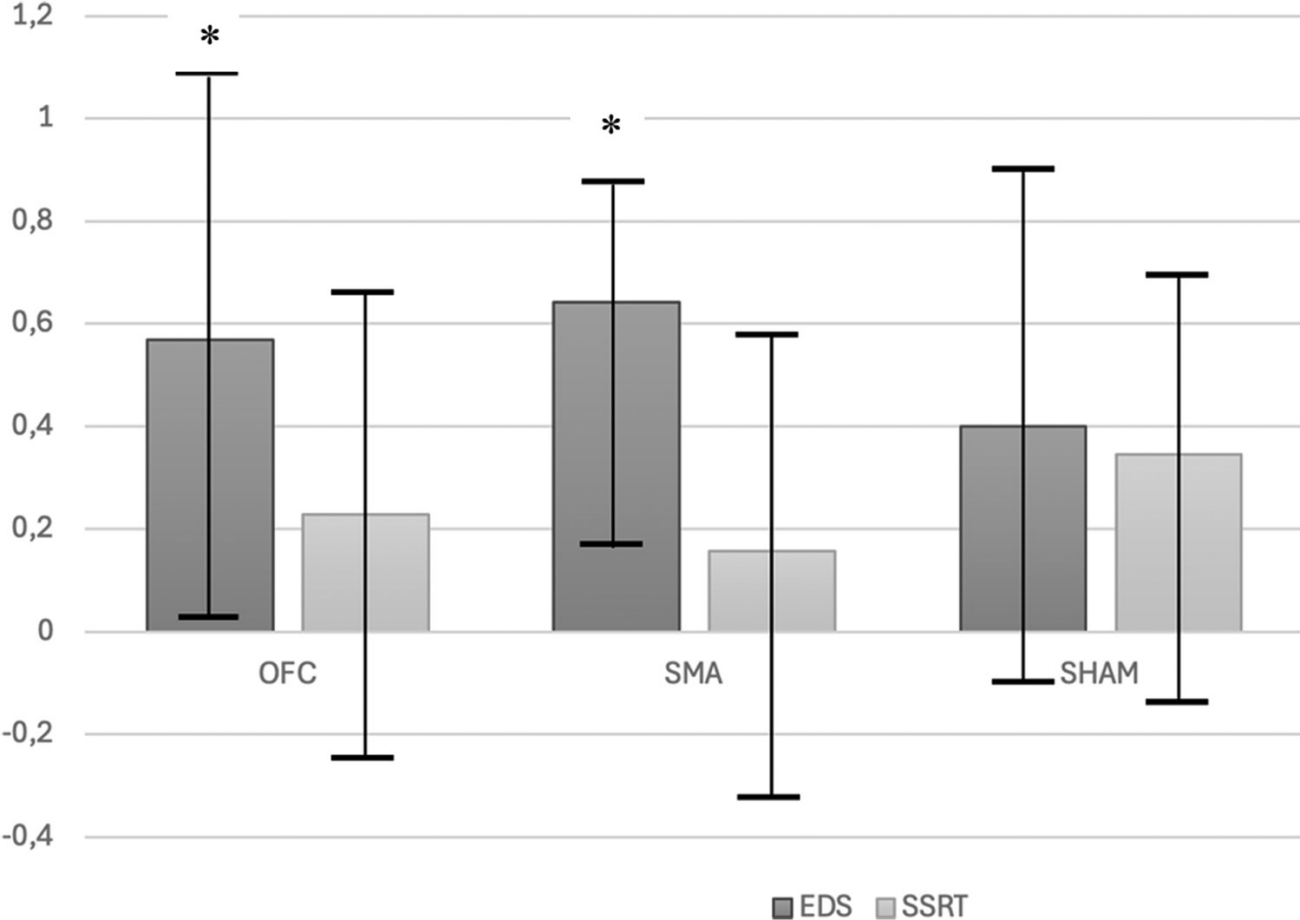
Effect sizes (95% CI) of the mean total number of extradimensional shift (EDS) errors and stop-signal reaction time (SSRT) following tDCS (pre–post effect size, baseline vs. 2-h after stimulation) for the three groups. CI, confidence interval; EDS, mean total number of extradimensional shift errors at stage 8 of the ID-ED task; ID-ED, intradimensional/extradimensional set shift task; SSRT, mean stop-signal reaction time (ms); tDCS, transcranial direct current stimulation; y-axis: effect sizes, corrected for nonnormality when needed. *Statistical significant pre–post improvement (*P* < 0.05) on the paired samples Student’s *t* test (normality could be assumed) or on the Wilcoxon signed-rank test (in cases where departures from normality were observed). L-OFC: *n* = 18; SMA: *n* = 19; Sham: *n* = 18.

Repeated multivariate analysis of variance did not detect significant differences between the three treatment arms in terms of cognitive performance at any stage of the study. In the within-group pre–post analyses (comparing baseline scores to scores 2-h after stimulation), we, however, found that tDCS of the L-OFC and SMA significantly improved cognitive inflexibility, measured as total errors on the EDS stage (stage 8) of the ID-ED task: for L-OFC, Student’s *t* = 2.27, *P* = 0.03, *d* = 0.57 (95% CI: 0.03 to 1.09); for SMA, Wilcoxon signed-rank test’s *z* = 2.19, *P* = 0.03, *r*_bs_=0.64 (95% CI: 0.18 to 0.87). Conversely, sham stimulation did not produce a significant improvement: Student’s *t* = 1.60, *P* = 0.131, *d* = 0.13 (95% CI: −0.12 to 0.90). No significant effect was found for tDCS on motor inhibition (SSRT) following any of the three treatment modalities (see Tables 1 and 2 and Fig. 1).

**Table 1:** Total number of extradimensional shift errors (intradimensional, extradimensional set shifting stage 8) and stop-signal reaction time scores (ms) at baseline and 2-h after stimulation of the left orbitofrontal cortex, supplementary motor area, or sham

	*n*	Mean	SD
L-OFC			
ID-ED – EDS, baseline	18	9.500	9.538
ID-ED – EDS, 2-h post	18	3.688	3.962
SST – SSRT, baseline	18	265.667	83.823
SST – SSRT, 2-h post	18	231.727	40.678
SMA			
ID-ED – EDS, baseline	19	9.105	9.427
ID-ED – EDS, 2-h post	19	4.625	6.781
SST – SSRT, baseline	19	265.137	81.513
SST – SSRT, 2-h post	19	249.482	61.763
Sham			
ID-ED – EDS, baseline	18	9.500	9.538
ID-ED – EDS, 2-h post	18	5.500	7.137
SST – SSRT, baseline	18	265.682	83.805
SST – SSRT, 2-h post	18	231.076	31.427

EDS, mean total number of extradimensional shift errors at stage 8 of the ID-ED task; ID-ED, intradimensional/extradimensional set shift task; L-OFC, left orbitofrontal cortex; SSRT, stop-signal reaction time; SST, stop-signal task.

**Table 2:** Pre–post stimulation effect sizes of changes in the mean total number of extradimensional shift errors and stop-signal reaction time following transcranial direct current stimulation targeting left orbitofrontal cortex, supplementary motor area, or sham

Task	L-OFC	SMA	Sham
EDS	Student’s *t* = 2.27 ***P* = 0.03** *d* = 0.57 CI = 0.03 to 1.1	Wilcoxon test’s *z* = 2.19 ***P* = 0.03** *r*_bs_ = 0.64 CI = 0.184 to 0.87	Student’s *t* = 1.60 *P* = 0.13 *d* = 0.399 CI = −0.117 to 0.903
SSRT	Wilcoxon test’s *z* = 0.83 *P* = 0.42 *r*_bs_ = 0.23 CI = −0.23 to 0.65	Wilcoxon test’s *z* = 0.60 *P* = 0.57 *r*_bs_ = 0.16 CI = −0.34 to 0.59	Wilcoxon test’s *z* = 0.83 *P* = 0.22 *r*_bs_ = 0.35 CI = −0.17 to 0.71

Bold indicates significant *P* values

*d*: Cohen’s *d*; *r*_bs_: matched rank biserial correlation; L-OFC: *n* = 18; SMA: *n* = 19; Sham: *n* = 18.

CI, confidence interval; EDS, mean total number of extradimensional shift errors at stage 8 of the ID-ED task; ID-ED, intradimensional/extradimensional set shift task; L-OFC, left orbitofrontal cortex; SMA, supplementary motor area; SSRT, stop-signal reaction time.

No significant correlations were found between the Y-BOCS and SST or ID-ED task measures at baseline. In addition, multivariate regression analyses did not find any significant results linking baseline cognitive scores or the pre–post changes in either cognitive score with the change in Y-BOCS scores after stimulation, at 24-h following the final stimulation of each modality (primary clinical outcome) or at 7-days poststimulation.

## Discussion

Whereas no differences in cognition were detected between the three stimulation groups, possibly due to the small sample size, the principal finding from our exploratory analysis was an improvement in cognitive inflexibility (as measured by the EDS on the ID-ED task), which was present following active stimulation of both the L-OFC and the SMA but not following sham stimulation. By contrast, the findings for the SSRT indicate that motor impulsivity was not significantly altered by any of the treatments.

Our analyses, despite their preliminary nature, could imply that there is scope for specifically ameliorating cognitive inflexibility, but not motor impulse control, by treating patients with OCD with tDCS. Another interpretation could be that tDCS had a more general positive cognitive effect on learning that allowed patients to improve their performance on the EDS with repetition of the task.

Some evidence of a learning effect is usually seen following sequential application of the ID-ED and the SSRT (Cacciamani *et al*., 2018), as evidenced by the small positive effect sizes produced in our study following sham stimulation (Table 1). The fact that significant poststimulation improvement, however, is not seen in the sham condition and only after active tDCS suggests that the cognitive effect is not simply a nonspecific learning effect linked to repeated testing. Moreover, the crossover design and the fact that post-tDCS improvement is seen on the ID-ED task only hint to the possibility that the cognitive effect of tDCS is relatively specific in terms of improving flexibility. The 28 days of washout was sufficient to minimize carryover effects in terms of clinical changes following the different tDCS sessions (Fineberg *et al*., 2023). We, however, cannot conclusively rule out the possibility of a carryover effect on cognitive changes. Future studies adopting alternative parallel versions of cognitive tests and extended washout periods between tDCS sessions could help mitigate these potential biases.

While the patients in our study improved symptomatically on the Y-BOCS (Fineberg et al, 2023), we did not find a significant relationship between the magnitude of improvement in EDS and improvement on Y-BOCS, hence we are unable to link the changes seen in cognition with improvement in symptom scores, again probably due to the small sample size of our study.

Cognitive inflexibility may serve as a technique to mitigate the pathological uncertainty or doubt that constitutes a debilitating symptom of OCD (Marzuki *et al*., 2021; Cobos *et al*., 2022). The lack of correlation between cognitive inflexibility and symptom severity in OCD, illness duration, or treatment history, however, raises questions regarding this interpretation and reinforces its classification as a trait rather than a state marker (Robbins *et al*., 2019; Clarke *et al*., 2024), with its presence thought to adversely affect treatment outcomes. Some evidence suggests that cognitive inflexibility adversely affects the therapeutic efficacy of CBT in OCD, while its influence on the responsiveness to SSRIs remains less clear (D’Alcante *et al*., 2012; Simpson *et al*., 2021). Other research indicates that individuals with OCD who exhibit rigidity and obstinacy tend to be more resistant to treatment overall (Wetterneck *et al*., 2011) and face an increased chance of symptomatic recurrence. Indeed, a naturalistic prospective study of individuals with OCD revealed that participants with concomitant obsessive-compulsive personality disorder (OCPD), which is characterized by inflexible traits, were almost twice as likely to relapse over a 5-year follow-up period (*P* < 0.005) (Eisen *et al*., 2013). Another prospective naturalistic longitudinal study of patients with severe OCD, conducted during the COVID-19 pandemic (January 2019 to June 2021), published in poster format, indicated inferior clinical outcomes and an increased risk of symptomatic relapse in individuals with comorbid OCPD treated with combinations of medication, CBT, and social care (Cirnigliaro *et al*., 2021). Nevertheless, further study is needed to conclusively ascertain the impact of objectively assessed cognitive inflexibility as demonstrated using a cognitive task and clinical outcomes in OCD as well as in other mental disorders.

Our findings are interesting, as previous research has supported the classification of cognitive inflexibility as a latent trait marker of OCD, untouched by established treatments (Clarke *et al*., 2024). Indeed, our own work has shown that neither SSRI nor CBT with exposure response prevention – the two established evidence-based treatments for OCD – improves EDS in OCD (Reid *et al*., 2024). Hence, neurostimulation may act differently from established treatments for OCD with the advantage of improving aspects of disorder-related cognition. Our findings contrast with the recent meta-analysis by Clarke *et al*., (2024), in which cognitive inflexibility appears unrelated to treatment status; the reason for this discrepancy is unclear although it is possibly due to the fact that no study adopting noninvasive neurostimulation techniques provided data on cognitive flexibility in OCD and was included in the aforementioned meta-analysis. The lack of a correlation between cognitive changes and obsessive-compulsive symptoms, as measured by the Y-BOCS, and the fact that we did not find improvements in cognitive flexibility to predict these clinical outcomes may be explained by the size of our sample; however, it also is plausible that cognitive inflexibility exists as an orthogonal component independent from core OCD symptoms, in accordance with the meta-analysis by Clarke *et al*., (2024). Further well-powered investigations should be conducted to disentangle this important matter.

The effect of tDCS on cognitive flexibility might be explained through as yet unconfirmed neurobiological mechanisms, including modulation of neuronal excitability and promotion of synaptic plasticity. tDCS influences the polarization of neuronal membranes, leading to increased firing rates and enhanced synaptic strength, which are essential for adaptive cognitive strategies (Das *et al*., 2016). Research demonstrates that stimulation of the OFC can improve the brain’s ability to reconfigure existing neural pathways and form new connections, facilitating faster cognitive shifts in response to changing environments (Parmar *et al*., 2021). Additionally, the effects of tDCS extend to enhancing the connectivity of the OFC with other regions involved in cognitive control and executive functions, such as the dorsolateral prefrontal cortex (dlPFC), further contributing to improved cognitive flexibility (Soyata *et al*., 2019).

While cognitive flexibility improved under tDCS, no significant effects, however, were observed for motor impulsivity. Studies have tested the impact of tDCS on aspects of response inhibition in healthy individuals and found evidence of improvement, in particular in those studies targeting the dlPFC or the pre-SMA (Teti Mayer *et al*., 2020). In the study by Hsu *et al*. (2011), the authors showed that anodal tDCS over the pre-SMA improved inhibitory control as measured by the SST, while cathodal tDCS impaired it. The absence of a clinically significant effect in response inhibition in our study may therefore be explained by the protocol adopted (use of cathodal tDCS) or the small size of the sample. Another possible explanation could be the fact that other, as yet unidentified neural factors related to the presence of OCD interfere with the remediative effect of tDCS on motor disinhibition. Future studies, employing a larger sample size and adopting cathodal and anodal stimulations of the SMA are warranted to clarify this aspect.

A few small-scale trials have been conducted to examine the effects of different pharmacological agents on cognitive inflexibility. These agents include SSRI (Brigman *et al*., 2010), levodopa (Cools *et al*., 2003), the *N*-methyl-d-aspartic acid receptor antagonist ketamine in animal models (Nikiforuk and Popik, 2014), and memantine in humans (Grant *et al*., 2010). Additionally, the 5HT_2A_ agonist psilocybin has also been investigated (de Veen *et al*., 2017). These studies have shown promising signs of potential improvements in cognitive inflexibility; however, the evidence remains limited.

More compelling evidence, perhaps, derives from a few published translational studies that have investigated different components of the cortico-striato-thalamo-cortical (CSTC) pathways implicated in the development of OCD and related disorders (Calzà *et al*., 2019; Robbins *et al*., 2019) as potential interventional targets for improving cognitive inflexibility. A mechanistic study in patients with OCD showed that deep brain stimulation (DBS) targeting the antero-medial subthalamic nucleus, along with its cortical connections including the L-OFC, the dorsal portion of the anterior cingulate cortex (dACC), and the dlPFC, resulted in improvement in cognitive inflexibility as measured by the EDS, as well as a reduction in symptom severity as measured by the Y-BOCS (Goodman *et al*., 1989). These findings suggest that modulating CSTC ‘cognitive control’ circuits using DBS may be particularly beneficial for individuals with OCD and cognitive inflexibility (Tyagi *et al*., 2019, 2022). Taken together with the findings of this study, they also imply that noninvasive modulation of a cortical node, that is, the L-OFC, within the same cognitive loop, produces a similar beneficial effect. Substantiation of our finding in a larger, adequately powered study would have major clinical implications, as tDCS could feasibly be upscaled as a treatment to reach a considerable proportion of patients.

Other studies have investigated the effects of noninvasive forms of neurostimulation on other aspects of inflexible thinking. Asgharian Asl and Vaghef, (2022) found that rTMS of the left dlPFC, which has known antidepressant and anticompulsive effects (Pellegrini *et al*., 2022), was effective in improving depressive symptoms and cognitive measures related to disinhibition and inflexibility in female patients with depression. Other investigations into the effects of deep transcranial magnetic stimulation (TMS) targeting the ACC in patients with OCD, as reported by Carmi *et al*. (2019) and Luo *et al*. (2023), have demonstrated improvements in OCD symptoms accompanied by changes in electroencephalogram (EEG) cognitive control markers including event-related potentials known to be related to OCD, such as the error-related negativity, which is enhanced in OCD (de Souza, 2021; Lawler *et al*., 2021). Another randomized controlled trial of patients with OCD by Balzus *et al*., (2022), found that tDCS targeting the pre-SMA reduced the amplitude of the error-related negativity compared with sham (marginally significant). In addition, an open-label study in a relatively small sample of patients with OCD, which investigated the efficacy of tDCS applied to the right OFC alongside effects on cortical excitability and inhibition as measured by concurrent TMS-electroencephalography, found that tDCS was associated with both a significant improvement in Y-BOCS scores and a concurrent decrease in the amplitude of TMS-evoked N100 response – an EEG measure believed to be associated with GABA_B_ receptor activity. The authors suggested that tDCS administered to the OFC has the potential to mitigate the aberrant functioning of GABA_B_ receptors in OCD (Cheng *et al*., 2022).

## Limitations

While our study found within-group pre–post significant differences in the univariate analysis, with improvements in cognitive flexibility, the same result was not obtained in the multivariate analysis. As such, we have not identified a treatment-by-time interaction effect. This might be explained by the modest size of our sample and should be considered a limitation. The lack of an association between improvement in cognitive flexibility and obsessive-compulsive symptoms can also probably be explained by our sample size. Moreover, we should acknowledge the short duration of the postbaseline assessment period as another limitation. We did not control for the impact of medication, and heterogeneity in medication could represent yet another confounding factor; further research should aim to take into account and weigh the use of different pharmacotherapies that could have an influence on cognition.

## Future directions

Our results, therefore, can be seen to add to the growing literature showing encouraging findings regarding the use of noninvasive neurostimulation using tDCS targeting the OFC and the SMA in OCD (Bation *et al*., 2019; Gowda *et al*., 2019; Yoosefee *et al*., 2020; Silva *et al*., 2021) and, for the first time, provide preliminary evidence that tDCS can possibly improve inflexibility as measured by the EDS in OCD. One of the main advantages of tDCS over TMS is the low cost and ease of widespread dissemination, which would allow a more feasible clinical implementation.

Future studies should aim to recruit a larger sample and include longer-term follow-up assessments (e.g. 1 week, 1 month poststimulation) to increase statistical power and allow for more robust conclusions. This would also enable subgroup analyses (e.g. medication-naive vs. medicated patients) to better understand the effects of tDCS and provide valuable information on the durability of tDCS effects on cognition and symptoms. Further research is needed to explore the relationship between cognitive improvements and clinical outcomes in more detail and to investigate other cognitive domains (e.g. working memory, attention) to provide a more comprehensive understanding of the effects of tDCS in OCD. Our study highlights the variability in tDCS protocols across the literature; future works should aim to standardize tDCS parameters (e.g. intensity, duration, electrode placement) to facilitate comparisons across studies and enhance reproducibility.

If our findings were to be replicated in larger cohorts, with evidence of differences between active and sham treatment arms, tDCS targeting the L-OFC or SMA could potentially constitute an effective and feasible intervention for use in precision medicine and personalized care approaches. For example, tDCS to the OFC or SMA may be particularly suitable for subgroups known to have a high degree of inflexibility, such as those with comorbid OCPD (Fineberg *et al*., 2015). Moreover, cognitive testing could potentially be used at screening to detect those who may benefit from tDCS most, or during treatment to detect cognitive improvements, which may appear rapidly before the symptomatic improvement is present to a measurable degree and which may augur a good clinical outcome.

## Conclusions

Cognitive inflexibility has been shown to be a challenge in clinical practice, but to date, no specific intervention has been found to improve this area of dysfunction in OCD (Chamberlain, Fineberg, *et al*., 2006; Vriend *et al.*, 2013; Reid *et al*., 2024). Based on the findings of this small double-blind trial, our findings suggest that tDCS targeting the L-OFC or the SMA represents a feasible and potentially effective option for treating cognitive inflexibility in patients with OCD and that the brain regions associated with cognitive flexibility may be particularly responsive to tDCS treatment.

## Acknowledgements

The present research was jointly funded by the University of Hertfordshire and the Hertfordshire Partnership Foundation NHS Trust.

### Conflicts of interest

There are no conflicts of interest.
